# Functional characterisation of *cis*-regulatory elements governing dynamic *Eomes* expression in the early mouse embryo

**DOI:** 10.1242/dev.147322

**Published:** 2017-04-01

**Authors:** Claire S. Simon, Damien J. Downes, Matthew E. Gosden, Jelena Telenius, Douglas R. Higgs, Jim R. Hughes, Ita Costello, Elizabeth K. Bikoff, Elizabeth J. Robertson

**Affiliations:** 1 The Sir William Dunn School of Pathology, University of Oxford, Oxford OX1 3RE, UK; 2 MRC Molecular Haematology Unit, Weatherall Institute of Molecular Medicine, University of Oxford, John Radcliffe Hospital, Oxford OX3 9DS, UK

**Keywords:** Eomesodermin, Enhancer, Capture-C, Nodal signalling, Definitive endoderm

## Abstract

The T-box transcription factor (TF) Eomes is a key regulator of cell fate decisions during early mouse development. The *cis*-acting regulatory elements that direct expression in the anterior visceral endoderm (AVE), primitive streak (PS) and definitive endoderm (DE) have yet to be defined. Here, we identified three gene-proximal enhancer-like sequences (PSE_a, PSE_b and VPE) that faithfully activate tissue-specific expression in transgenic embryos. However, targeted deletion experiments demonstrate that PSE_a and PSE_b are dispensable, and only VPE is required for optimal *Eomes* expression *in vivo*. Embryos lacking this enhancer display variably penetrant defects in anterior-posterior axis orientation and DE formation. Chromosome conformation capture experiments reveal VPE-promoter interactions in embryonic stem cells (ESCs), prior to gene activation. The locus resides in a large (500 kb) pre-formed compartment in ESCs and activation during DE differentiation occurs in the absence of 3D structural changes. ATAC-seq analysis reveals that VPE, PSE_a and four additional putative enhancers display increased chromatin accessibility in DE that is associated with Smad2/3 binding coincident with transcriptional activation. By contrast, activation of the *Eomes* target genes *Foxa2* and *Lhx1* is associated with higher order chromatin reorganisation. Thus, diverse regulatory mechanisms govern activation of lineage specifying TFs during early development.

## Abstract (precis)

**Summary:** Expression of the mouse T-box factor Eomes is controlled by a key gene-proximal enhancer-like element, with changes in chromatin accessibility influencing its activity in definitive endoderm.

## INTRODUCTION

Reciprocal signalling cues between the pluripotent epiblast and adjacent tissues, namely the extra-embryonic ectoderm (ExE) and visceral endoderm (VE), precisely coordinate cell fate decisions during gastrulation. Nodal/Smad signals from the epiblast are required for specification of the AVE, a discrete signalling centre that establishes anterior-posterior (A-P) polarity (Brennan et al., 2001; Robertson, 2014; Stower and Srinivas, 2014). The A-P axis initially becomes visible at gastrulation, when proximal posterior cells undergo an epithelial-to-mesenchymal transition (EMT) at the PS to form nascent mesoderm. Slightly later, following distal extension of the streak, endoderm progenitors delaminate and emerge onto the surface of the embryo (Kwon et al., 2008).

The T-box transcription factor (TF) eomesodermin (Eomes), acting downstream of Nodal/Smad signals, is required to promote AVE formation and orientation of the A-P axis (Arnold et al., 2008a; Ciruna and Rossant, 1999; Nowotschin et al., 2013), as well as EMT of nascent mesoderm cells (Arnold et al., 2008a; Costello et al., 2011; Russ et al., 2000; van den Ameele et al., 2012). At post-implantation stages, *Eomes* is expressed in the ExE and embryonic-VE, robustly induced at the onset of gastrulation in the PS and maintained in the anterior PS as it extends, before being abruptly lost (coincident with node formation) (Kwon and Hadjantonakis, 2007). Fate-mapping experiments demonstrate that transient *Eomes* expression marks progenitors of the cardiovascular lineage, definitive endoderm (DE), node and midline (Costello et al., 2011).

Transgenic and targeted deletion approaches have provided insight into cell type-specific developmental enhancers that govern expression of key genes responsible for partitioning the pluripotent epiblast into discrete cell lineages. Proximal *cis*-regulatory regions within 20 kb of the transcriptional start sites (TSS) directing spatiotemporally restricted expression of *Nodal*, *Mesp1/2* and *Lhx1* have been identified. Both the ASE, an intronic autoregulatory enhancer (Adachi et al., 1999; Norris and Robertson, 1999), and the Wnt signalling responsive 5′ PEE (Ben-Haim et al., 2006) cooperatively regulate *Nodal* expression. Mutant embryos lacking these genomic sequences display dose-dependent defects in specification of mesoderm and DE/midline progenitors (Norris et al., 2002; Vincent et al., 2003). Similarly, the *Mesp1/2* genes, which are essential for formation of nascent mesoderm, are jointly regulated by the EME, an Eomes-dependent enhancer (Costello et al., 2011; Haraguchi et al., 2001). Our recent work demonstrates that *Lhx1*, which is required for AVE and anterior mesendoderm specification (Barnes et al., 1994; Shawlot and Behringer, 1995), is directly controlled by Eomes binding to a proximal promoter element (Nowotschin et al., 2013).

*Eomes*, which is rapidly induced in the proximal-posterior epiblast coincident with the acquisition of A-P polarity (Ciruna and Rossant, 1999), is widely viewed as a master regulator of mesendodermal lineages (Costello et al., 2011; Izumi et al., 2007; Teo et al., 2011; van den Ameele et al., 2012). Thus, *Eomes* represents the earliest lineage-specifying gene in the embryo proper. However, relatively little is known about the *cis*-acting regulatory elements controlling its dynamic pattern of expression. Recent studies of mouse and human ESCs have identified a conserved switch enhancer −7 kb upstream of the TSS (Beyer et al., 2013; Kartikasari et al., 2013; Rada-Iglesias et al., 2011) that is repressed under self-renewing conditions (Teo et al., 2011), and becomes activated during mesoderm and endoderm differentiation. However, possible functional contributions made by this genomic region have yet to be assessed *in vivo*.

Here, we investigate the structural features of the locus that govern *Eomes* expression during early mouse development. Gain-of-function transgenic reporter assays identified three gene-proximal *Eomes* enhancer-like sequences (PSE_a, PSE_b and VPE). However, when we engineered germline deletions to evaluate their functional contributions *in vivo*, surprisingly, only the VPE was found to influence expression in the early embryo. We also exploited Next Generation (NG) Capture-C technology (Davies et al., 2016) to describe the 3D structural features of the locus. The *Eomes* promoter occupies a discrete 500 kb regulatory compartment in ESCs, and this chromatin conformation is not appreciably altered during DE differentiation. However, our ATAC-seq analysis revealed that the VPE, PSE_a and four additional distal regulatory elements located within this pre-formed compartment display increased chromatin accessibility and acquire Smad2/3 occupancy during DE differentiation. This mode of 3D genome organisation probably serves to facilitate rapid Nodal/Smad-dependent activation of the locus. By contrast, developmentally regulated *Foxa2* and *Lhx1* promoter-promoter and promoter-enhancer interactions seem to require substantial structural changes during the shift from a transcriptionally inactive to active conformation.

## RESULTS

### Identification of proximal *Eomes* enhancers that are active during gastrulation

Putative enhancer elements containing DNase I hypersensitive sites and marked by H3K4me1 are considered to be active if also enriched for H3K27ac or, alternatively, viewed as poised if enriched for H3K27me3 (Rada-Iglesias et al., 2011; Zentner et al., 2011). To identify candidate enhancers at the *Eomes* locus, we examined ChIP-seq datasets from undifferentiated ESC, epiblast-like cells (EpiLC) and mesodermal precursors (MES) (Alexander et al., 2015; Buecker et al., 2014; ENCODE Project Consortium, 2012) corresponding to the E4.5 epiblast (ESC), the E5.5 epiblast (EpiLC) or E6.5 primitive streak (MES) cell populations.

**Fig. 1.**
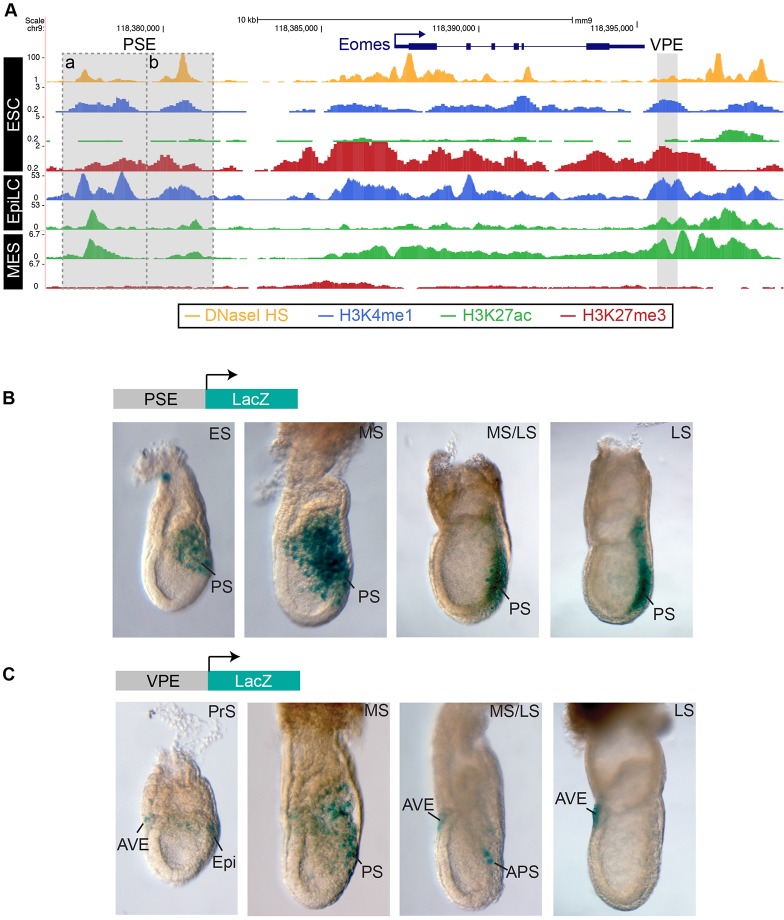
**Mapping proximal *Eomes* enhancers active at gastrulation.** (A) ChIP-seq of H3K4me1, H3K27me3 and H3K27ac, and DNaseI hypersensitivity (HS) in ESCs, epiblast-like cells (EpiLC) and mesoderm (MES) (Alexander et al., 2015; Buecker et al., 2014; ENCODE Project Consortium, 2012) identify potential proximal *Eomes* enhancers that are activated during differentiation. The PSE cluster and VPE regions are highlighted in grey. (B,C) X-gal-stained transgenic embryos expressing enhancer-driven *LacZ* reporters. (B) PSE reporter activity is confined to the primitive streak (PS) at early- (ES), mid- (MS) and late-streak (LS) stages of gastrulation (2/4 transgenic mouse lines). (C) VPE reporter activity detectable in the proximal posterior epiblast (Epi) at the pre-streak (PrS) stage and in the PS at the MS stage, becomes restricted to the anterior PS (APS) and is lost at LS stage. Between the PrS stage and the LS stage, VPE activity is also detectable in the anterior visceral endoderm (AVE) (2/6 transgenic mouse lines).

We identified three DNase I hypersensitive sites close to the *Eomes* promoter marked by H3K4me1 that show increased H3K27ac upon differentiation, including two sites (PSE_a and PSE_b) located close together, spanning a 5 kb region between −11 kb to −6 kb upstream of the transcriptional start site (TSS), and a third candidate region (VPE) lying +8 kb downstream of the TSS (Fig. 1A, Fig. S1A). Notably, the upstream cluster contains the previously described switch enhancer (PSE_b) activated during ESC differentiation to DE and mesendoderm (Beyer et al., 2013; Kartikasari et al., 2013). Additionally, two downstream DNaseI hypersensitive sites bound by CCCTC-binding factor (CTCF) were identified in ESCs (Fig. S1A). The three proximal regions are highly conserved among mammals (Fig. S1A), associated with H3K4me1/H3K27me3 in ESCs and, thus, probably represent poised enhancers that are primed for activation. Consistent with a shift to the active state during the transition from pluripotency to lineage commitment, these regions contain increased H3K27ac and decreased H3K27me3 in EpiLC and MES. The homologous regions are also associated with active enhancer marks in human DE cultures (Fig. S1B).

To test the activities of these candidate enhancers, we generated transgenic strains carrying *LacZ* reporter constructs and subsequently examined embryonic expression at early post-implantation stages (Kothary et al., 1989). The 5 kb upstream region was designated the PSE (primitive streak enhancer) because PSE-*LacZ* activity is restricted to the PS at early (ES), mid- (MS) and late-streak (LS) stages (Fig. 1B). There was no detectable *LacZ* expression in the ExE or VE. However, the 0.7 kb downstream enhancer, designated the VPE (visceral endoderm and primitive streak enhancer), showed activity in the proximal-posterior epiblast, and also in the AVE at pre-streak (PrS) stages (Fig. 1C). Slightly later, *LacZ* expression was detectable in the PS, nascent mesendoderm and the AVE, subsequently became restricted to the anterior PS, and was lost by LS stages. Collectively, these three enhancers faithfully recapitulate the endogenous *Eomes* expression patterns within both the VE and embryo proper.

### The PSE is dispensable for normal embryonic development

**Fig. 2.**
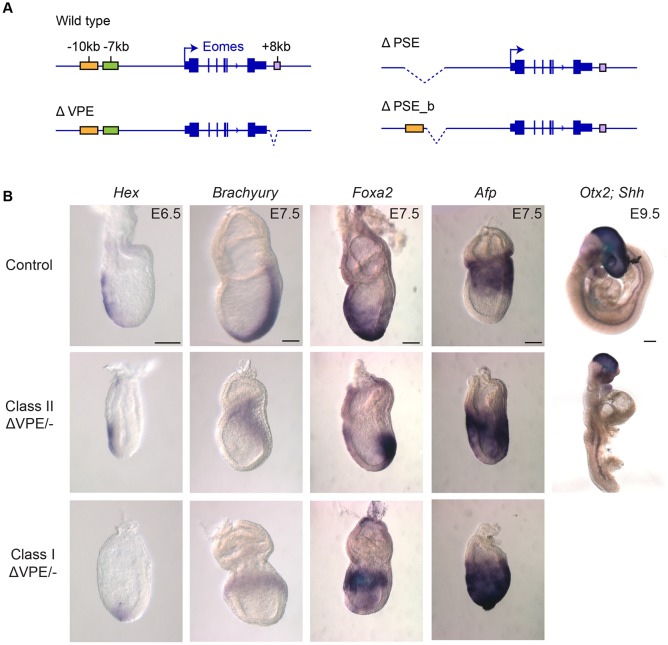
**Targeted deletions of proximal enhancers show that only the VPE is required for proper gastrulation.** (A) Targeted deletions of the 5 kb ΔPSE, 2 kb ΔPSE_b and 0.7 kb ΔVPE generated by homologous recombination (Figs S2-S4). (B) Whole-mount *in situ* hybridisation of *Eomes^ΔVPE/−^* embryos. Class I mutants exhibit failure in A-P axis specification; class II display APS defects. At E6.5 in class I mutants, expression of the AVE marker *Hex* is confined to the distal VE (*n*=4/10 *Eomes^ΔVPE/−^* embryos analysed). At E7.5, the mesoderm marker *Brachyury* (*n*=2/5) and the DE marker *Foxa2* (*n*=3/7) are mislocalised proximally. In class II mutants, *Hex* marks the AVE, *Brachyury* expression fails to extend distally (*n*=3/5), whereas the *Foxa2* domain is confined to the APS and the DE domain is lost (*n*=3/7). Consistent with failure to specify DE in both mutant classes, expression of *Afp*+ VE cells fails to disperse proximally (for class I and class II, *n*=2 and *n*=2 out of 7 *Eomes^ΔVPE/−^* embryos analysed, respectively). At E9.5, class II mutants display venture closure and neural tube defects, fused or malformed somites, loss of *Otx2*+ forebrain tissue and an anterior truncation of the *Shh* midline (*n*=3/3 viable morphologically abnormal *Eomes^ΔVPE/−^* embryos recovered). Scale bars: 100 μm.

The 5 kb PSE contains both an upstream element, PSE_a, as well as the previously described PSE_b switch enhancer reported to interact with the *Eomes* promoter during DE differentiation (Fig. S1A) (Beyer et al., 2013; Kartikasari et al., 2013). To investigate their functional activities in the context of the developing embryo, we generated discrete germline targeted deletions (Fig. 2A, Fig. S2). Surprisingly, homozygous mice lacking the 2 kb PSE_b genomic fragment ∼8 kb to ∼6 kb upstream of the TSS (ΔPSE_b) were recovered at Mendelian ratios and are indistinguishable from wild-type littermates (Table 1A). These results demonstrate that the PSE_b is dispensable *in vivo*. It is well known that heterozygous mice carrying null alleles (*Eomes*^GFP/+^, *Eomes*^LacZ/+^ or *Eomes*^Δexon2-5/+^) are fully viable (Arnold et al., 2008a, 2009; Russ et al., 2000). To investigate whether the PSE_b deletion may compromise transcriptional output, we crossed *Eomes*^ΔPSE_b/ΔPSE_b^ mice to those carrying the *Eomes*^GFP/+^ allele (hereafter referred to as *Eomes* null; *Eomes*^+/−^). The resulting *Eomes*^ΔPSE_b/−^ compound mutants develop normally (Table 1B).

**Table 1.**
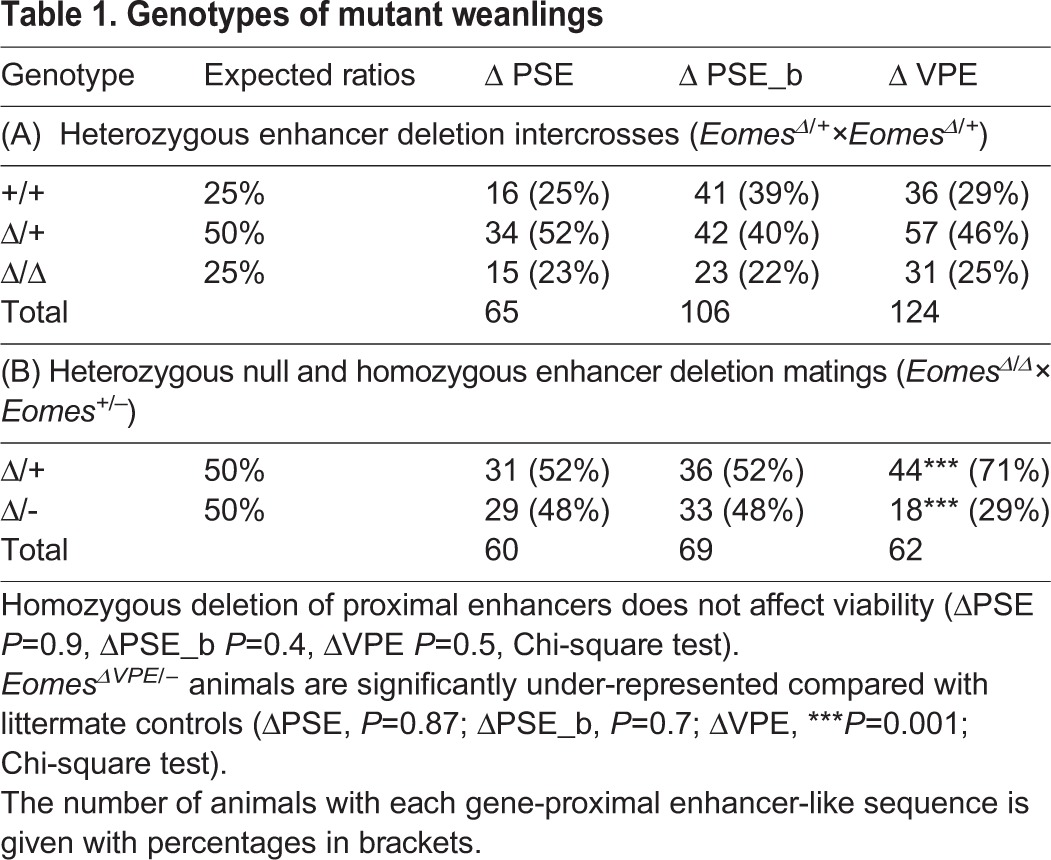
**Genotypes of mutant weanlings**

Next, we engineered a deletion that eliminates the entire 5 kb PSE cluster (referred to as ΔPSE, Fig. S3). However, as for the PSE_b, removal of the entire PSE region in *Eomes*^ΔPSE/ΔPSE^ mice has no noticeable effect on viability (Table 1A). Finally, crossing these deletion mutants with mice carrying the *Eomes* null allele also failed to perturb embryonic development (Table 1B). Thus, it appears that the PSE can activate expression in gain-of-function transgenic embryos. Nonetheless, this genomic region is clearly dispensable for *Eomes* expression *in vivo*.

### Targeted deletion of the VPE leads to defective gastrulation

To investigate functional contributions made by the VPE, we generated a targeted deletion lacking this 0.7 kb region (Fig. S4). Homozygous ΔVPE mutants are viable and fertile (Table 1A). However, when we crossed *Eomes*^ΔVPE/ΔVPE^ mice with *Eomes*^+/−^ heterozygous animals carrying the null allele, we observed a significant under-representation of viable *Eomes*^ΔVPE/−^ compound heterozygotes (Table 1B), with ∼40% (*n*=18) of the expected numbers recovered at weaning (equivalent to *Eomes*^ΔVPE/+^, *n*=44). These results strongly suggest that *Eomes*^ΔVPE^ acts as a hypomorphic allele.

Next, to determine the onset of lethality, we examined embryos from E6.5 onwards. Approximately one-third of *Eomes*^ΔVPE/−^ embryos are morphologically normal. However, two distinct classes of abnormal embryos were recovered at roughly equivalent numbers. The most severely affected (class I) mutants arrest at early gastrulation stages, while a second group (class II) progress to mid-gestation (Fig. 2B).

In class I embryos, the AVE marker *Hex* is induced at E6.5 but remains localised to the distal tip. Thus, the AVE is specified but fails to migrate towards the prospective anterior side of the embryo. These embryos fail to correctly orient the A-P axis and lack a discrete PS. At E7.5, mesoderm (*Brachyury*) and DE (*Foxa2*) markers are restricted proximally. Class I mutant embryos, distinguished by the accumulation of disorganised mesenchymal cells in the epiblast cavity and a constriction at the embryonic and extra-embryonic boundary, phenocopy those selectively lacking *Eomes* activity in the VE (Nowotschin et al., 2013). Taken together with results above that demonstrate VPE-*LacZ* expression in the VE, the simplest explanation is that these abnormalities are caused by loss of *Eomes* function in the VE.

The class II embryos, which represent approximately one-third of the *Eomes*^ΔVPE/−^ embryos, successfully establish normal A-P polarity. However, as gastrulation proceeds they display focal defects in the anterior PS (APS) and its derivatives the DE, midline, node and notochord. Brachyury (*T*) expression in the PS fails to extend to the distal tip of the streak at E7.5. *Foxa2*-positive DE progenitors are specified but fail to migrate anteriorly. As judged by *Afp* expression, the VE is retained over the epiblast and fails to become distally restricted. These tissue disturbances probably reflect the functional loss of *Eomes* within the APS (Arnold et al., 2008a; Teo et al., 2011). APS derivatives are known to provide essential trophic signals required for patterning the anterior neurectoderm (Arkell and Tam, 2012). Consistent with this, at E9.5, class II mutant embryos display ventral closure and neural tube defects, fused or malformed somites, and loss of forebrain tissue.

### The VPE is required for optimal *Eomes* expression levels

**Fig. 3.**
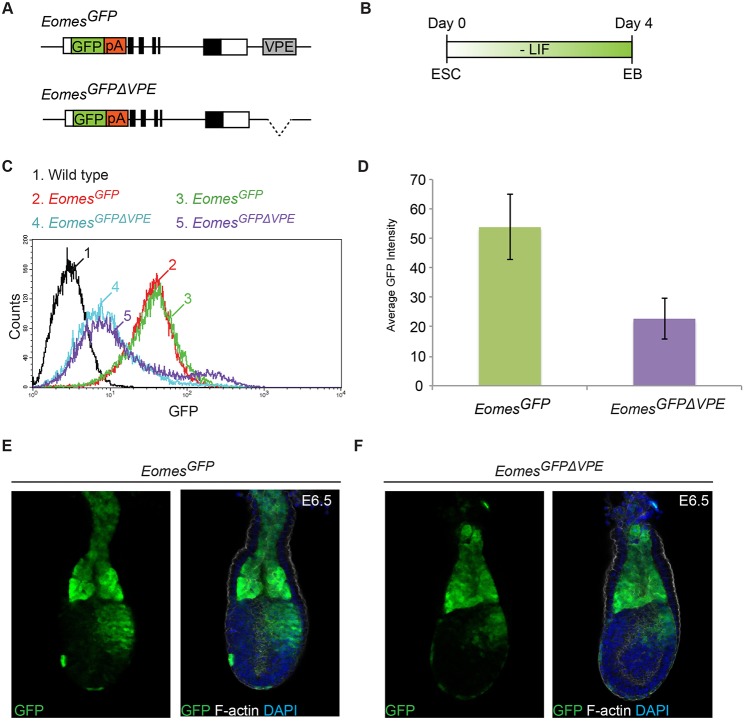
**VPE deletion profoundly reduces the level of *Eomes*^GFP^ reporter expression.** (A) Configuration of the *Eomes^GFP^* and *Eomes^GFPΔVPE^* alleles (Fig. S5). (B) Schematic of the embryoid body (EB) differentiation protocol. (C,D) Flow cytometry analysis of wild-type, *Eomes^GFP/+^* and *Eomes^GFPΔVPE/+^* day 4 EBs. (C) Representative histograms showing wild-type, two independently targeted *Eomes^GFP/+^* and two *Eomes^GFPΔVPE/+^* clones. (D) Average GFP intensity in *Eomes^GFP/+^* (*n*=4) and *Eomes^GFPΔVPE/+^* (*n*=4) cultures. Deletion of the VPE significantly reduces expression to 42% of the intact *Eomes^GFP^* reporter (*P*=0.05, Student's *t*-test). Error bars represent the s.e.m. (E,F) Confocal images of *Eomes^GFP^* and *Eomes^GFPΔVPE^* reporter expression in E6.5 embryos stained with anti-GFP antibody, DAPI (DNA) and phalloidin (F-actin). Domains of reporter expression are not perturbed by VPE deletion.

To test directly whether targeted loss of the VPE compromises *Eomes* transcriptional output, we eliminated the VPE in the context of our *Eomes*^GFP^ reporter allele containing an EGFP-pA cassette inserted in-frame at the translational start site in exon 1 (Fig. 3A, Fig. S5) (Arnold et al., 2009) and performed flow cytometry analysis to quantify expression levels. The *Eomes*^GFP^ reporter is robustly activated during ESC differentiation to embryoid bodies (EBs) (Costello et al., 2011) (Fig. 3B). As shown in Fig. 3C, GFP expression is dramatically reduced in *Eomes*^GFPΔVPE/+^ EBs when compared with *Eomes*^GFP/+^ EBs. The VPE deletion results in markedly reduced expression to 42% of the control *Eomes*^GFP/+^ EBs (Student's *t*-test *P*=0.05) (Fig. 3D).

These heterogenous EB cultures contain mixtures of cardiac mesoderm, DE and VE Eomes^+^ cell populations. To investigate the impact of the VPE deletion *in vivo*, we generated *Eomes*^GFPΔVPE/+^ mice and examined expression during gastrulation. GFP expression in *Eomes*^GFPΔVPE/+^ embryos recapitulates domains of the *Eomes*^GFP/+^ control embryos at E6.5, in the ExE, PS, nascent mesoderm and VE (Fig. 3E,F). The VPE deletion reduced expression levels but tissue-specific expression patterns were unperturbed. Similar conclusions were reached by whole-mount *in situ* hybridisation experiments examining *Eomes* mRNA expression in *Eomes*^ΔVPE/ΔVPE^ embryos (Fig. S4E). Thus, reduced *Eomes* transcription (∼50%) as in *Eomes*^+/−^ or *Eomes*^ΔVPE/ΔVPE^ embryos is sufficient to promote A-P axis specification and gastrulation. However, as shown above, further reduced expression (∼25%) in Eomes^ΔVPE/−^ embryos results in gastrulation defects.

### Foxh1-independent Nodal/Smad2/3 signals regulate VPE activity

**Fig. 4.**
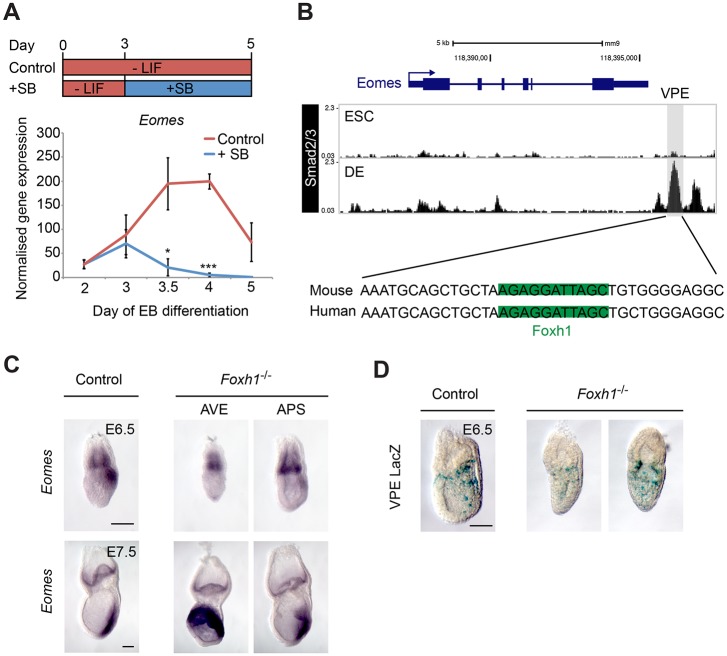
**VPE expression is regulated by Smad2 and independently of Foxh1.** (A) RT-qPCR analysis of *Eomes* mRNA expression during EB differentiation. SB-431542 (SB) inhibition of Nodal/Smad2 signalling from day 3 onwards significantly reduces *Eomes* expression at d3.5 and d4 of differentiation (*P<0.05, ***P<0.001, Student's *t*-test, *n*=3). Error bars represent s.e.m. (B) ChIP-seq of Smad2/3 in definitive endoderm (DE) reveals binding to the VPE (Yoon et al., 2015), overlapping a predicted and conserved binding site for Foxh1, identified with JASPAR at >80% confidence (Mathelier et al., 2016). (C) Whole-mount *in situ* hybridisation of *Eomes* mRNA in control and *Foxh1*-null embryos. *Eomes* is expressed in both AVE- and APS-defective Foxh1 mutant subtypes at E6.5 and E7.5. (D) VPE-*LacZ* reporter activity both in the VE and epiblast is retained in *Foxh1* mutant embryos at E6.5. Scale bars: 100 μm.

*Eomes* activation in the VE and PS depends on Nodal/Smad signals (Brennan et al., 2001; Nowotschin et al., 2013). To investigate Nodal/Smad requirements in cultured EBs, we used the small molecule SB-431542 (SB), a potent inhibitor of type 1 activin receptor like kinases 4, 5 and 7. As expected, in control cultures, maximal *Eomes* expression was detectable between day (d)3.5 and d4 (Fig. 4A). *Eomes* expression was dramatically reduced in cultures treated with the SB inhibitor from d3, and by d4 is severely compromised to only 2% of that seen in controls (Fig. 4A). These results confirm that Nodal signalling is required to induce *Eomes* expression during the transition from pluripotency to lineage commitment. Additionally, when we compared Smad2/3 ChIP-seq datasets in ESC and DE cultures (Yoon et al., 2015), we found evidence for Smad2/3 occupancy at the VPE specifically in DE cultures (Fig. 4B). These observations strengthen the idea that Nodal/Smad signals controlling *Eomes* expression activate transcription via the VPE.

It is well known that the forkhead transcription factor Foxh1 functions as a Smad2/3 co-factor governing Nodal/Smad target gene expression (Attisano et al., 2001; Izzi et al., 2007). Foxh1 has been proposed to act as a pioneer factor and to recruit Smad2/3 complexes to switch enhancers, activated as ESCs transition to DE fates (Beyer et al., 2013; Cirillo et al., 2002; Cirillo and Zaret, 1999; Kim et al., 2011). Interestingly, the VPE Smad2/3 peak also contains a conserved Foxh1-binding motif. Moreover, the VPE region is co-bound by FOXH1, SMAD2/3 and SMAD4 in human DE cultures (Fig. S6) (Beyer et al., 2013; Brown et al., 2011; Kim et al., 2011; Teo et al., 2011). Consistent with the idea that Foxh1 cooperatively activates *Eomes* expression via the VPE, homozygous null *Foxh1*^−/−^ embryos phenocopy the *Eomes*^ΔVPE/−^ embryos, displaying either defective AVE formation prior to gastrulation or disturbances in APS specification at later stages (Hoodless et al., 2001; Yamamoto et al., 2001).

To evaluate directly *Foxh1* functional contributions, we analysed *Eomes* expression at E6.5 and E7.5 in the context of *Foxh1*^−/−^ mutant embryos (Fig. 4C). In mutants with AVE/DVE defects at E6.5, *Eomes* is expressed in the thickened VE at the distal tip of the embryo, and at E7.5 in the chorion and proximal epiblast. *Foxh1* mutants with APS defects express *Eomes* in the ExE and PS. *Eomes* is clearly expressed in both classes of *Foxh1* mutant embryos. Slightly reduced levels in the PS can be explained by the loss of Foxh1-dependent activation of the auto-regulatory ASE *Nodal* enhancer (Norris et al., 2002). In striking contrast to *Eomes/Nodal* double heterozygotes (Arnold et al., 2008a), we found no evidence here for *Eomes* and *Foxh1* genetic interactions. Indeed, *Eomes* and *Foxh1* compound mutant mice are fully viable (Table 2). Finally, to confirm that VPE activity is Foxh1 independent, we examined expression of the VPE-*LacZ* transgene in *Foxh1* mutant embryos. *LacZ* staining is detectable throughout the epiblast at E6.5 (Fig. 4E), and also in the thickened VE at the distal tip. Foxh1 function is nonessential for VPE-*LacZ* reporter activity. Thus, we conclude that Nodal/Smad signals activate *Eomes* expression in a Foxh1-independent manner, raising the possibility that other forkhead family members may recruit Smad2/3 complexes during Eomes induction *in vivo*.

**Table 2.**
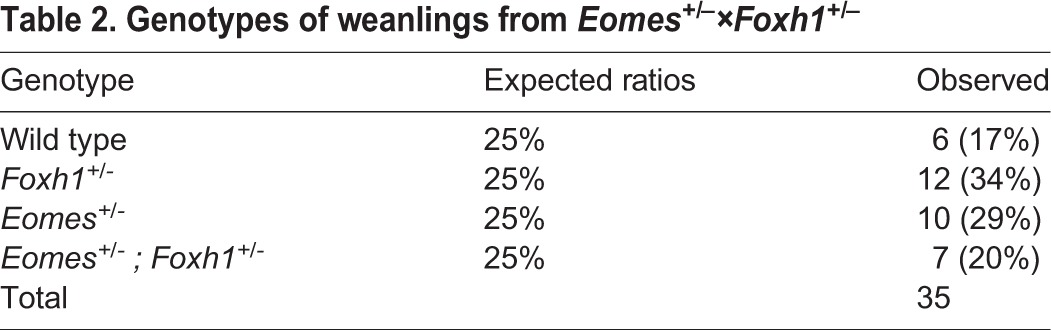
**Genotypes of weanlings from *Eomes^+/–^×Foxh1^+/–^***

### Characterisation of the *Eomes* 3D regulatory chromatin compartment during endoderm differentiation

The finding that the VPE targeted deletion partially reduces but fails to completely eliminate *Eomes* expression, strongly suggests that additional regulatory elements contribute to transcriptional output of the locus. Enhancer interactions with target promoters have been analysed by chromatin conformation capture techniques (de Wit and de Laat, 2012). We took advantage of the recently developed Next Generation (NG) Capture-C methodology (Davies et al., 2016) to screen for *Eomes* regulatory enhancer elements. During DE differentiation, *Eomes* expression increased by ∼600 fold (Fig. S7B) resulting in activation of the Eomes target genes, *Lhx1* and *Foxa2* (Fig. S7C) (Nowotschin et al., 2013; Teo et al., 2011).

NG Capture-C using viewpoints from the PSE_a and PSE_b exhibited promoter interactions in ESC (Fig. S8) when analysed with FourCseq (Klein et al., 2015). These interactions were marginally reduced in DE. However, the overall change was not statistically significant. By contrast, NG Capture-C revealed significant interactions between the VPE and the *Eomes* promoter in both ESC and DE cells (Fig. S8). Thus, the locus appears to be primed for activation prior to expression.

**Fig. 5.**
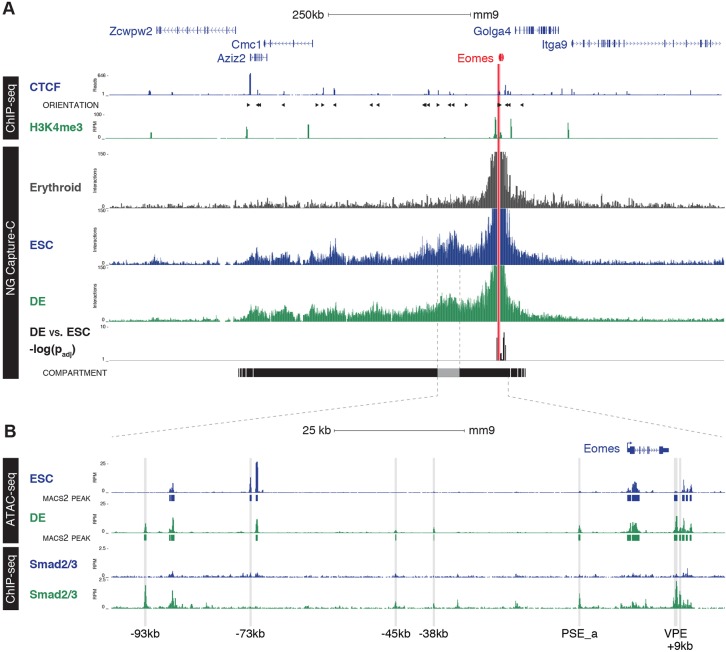
***Eomes* is regulated by Smad2/3 binding in a preformed compartment.** (A) NG Capture-C interaction profiles of the *Eomes* promoter (chr9:117,683,476-118,771,067) from erythrocytes (grey), ESC (blue) and DE (green). Tracks show mean interactions of normalised biological replicates (*n*=3) and DESeq2 significant differences between DE and ESC [−log(*P*_adj_); *P*≤0.05]. The *Eomes* compartment, as determined by boundaries of strong promoter interactions with CTCF orientation (arrowheads), is based upon binding in ESCs (Handoko et al., 2011). Histone modifications for H3K4me3 (DE, *n*=3) show promoter regions. (B) Enlargement of the region of the Eomes compartment showing highest association with the promoter, from chr9: 118,252,500-118,405,500. Open chromatin was generated using ATAC-seq in ESC and DE (*n*=3), with the addition of MACS2 called peaks annotated beneath each ATAC-seq track and Smad2/3 ChIP-seq in ESCs (blue) and DE (green) (Yoon et al., 2015). Regions of chromatin accessibility unique to ESCs (−73 kb) and those associated with Smad2/3 occupancy in DE (−93 kb, −45 kb, −38 kb, PSE_a, VPE and +9 kb) are indicated.

Next, performing Capture-C using a viewpoint from the *Eomes* promoter revealed that the *Eomes* locus, together with an upstream 300 kb gene desert and its neighbouring genes *Azi2* and *Cmc1*, occupies a discrete ∼500 kb chromatin compartment (Fig. 5A). This region contains numerous CTCF-binding sites (Handoko et al., 2011). Consistent with CTCF-mediated chromatin loops forming the compartment boundaries, motif analysis suggests that the outermost binding sites face inwards (Fig. 5A). This compartment structure is readily detectable in both ESC and DE cells but is completely absent in control terminally differentiated erythrocytes lacking *Eomes* expression (Fig. 5A, Fig. S9). Comparison of the NG Capture-C data from ESC and DE, in which the Eomes locus is transcriptionally silent or active, respectively, demonstrates that the compartment is highly stable. Moreover, there were no detectable changes in long-range promoter interactions within the compartment (Fig. S10).

To map changes in regions of open chromatin associated with *Eomes* activation and identify potential novel DE enhancers within the compartment, we performed ATAC-seq. We identified 85,581 total peaks in ESC and DE, and of these 19% were gained and 32.5% lost during differentiation (Fig. S9). Within the *Eomes* compartment we identified six regions that show increased accessibility in DE, including the VPE and the PSE_a, as well as four additional sites at −93 kb, −45 kb, −38 kb and +9 kb relative to the *Eomes* TSS (Fig. 5B).

Next, we examined Smad2/3 binding across the compartment (Yoon et al., 2015). Smad2/3 occupancy was detectable in DE but not in ESCs at all six of the differentially accessible sites (Fig. 5B). These findings demonstrate the *Eomes* locus is organised into a large 3D regulatory chromatin compartment in pluripotent ESCs that is maintained upon DE differentiation. Global structural changes are not required for *Eomes* induction during DE differentiation. Rather, transcriptional activation seems to reflect increased chromatin accessibility and Smad2/3 recruitment at DE enhancers. The −95 kb and −45 kb regions, and to a lesser extent the −38 kb region, are associated with poised and active enhancer marks as cells transition from ES to Epi to MES states, respectively (Fig. S11). Additionally, recently published TF ChIP-seq data demonstrate that the −45 kb ATAC-seq peak, together with the PSE_a and VPE, are co-bound by Tcf3 in DE (Wang et al., 2017), suggesting that both Nodal and Wnt signalling converge on these enhancer regions during gastrulation (Ben-Haim et al., 2006). Consistent with its activities as a key *Eomes* regulatory element during DE specification, the VPE is also bound by Otx2 and Lhx1 in EpiLC and mesendoderm cultures, respectively (Buecker et al., 2014; Costello et al., 2015).

### *Foxa2* and *Lhx1* promoters form long-range interactions in polycomb bodies

**Fig. 6.**
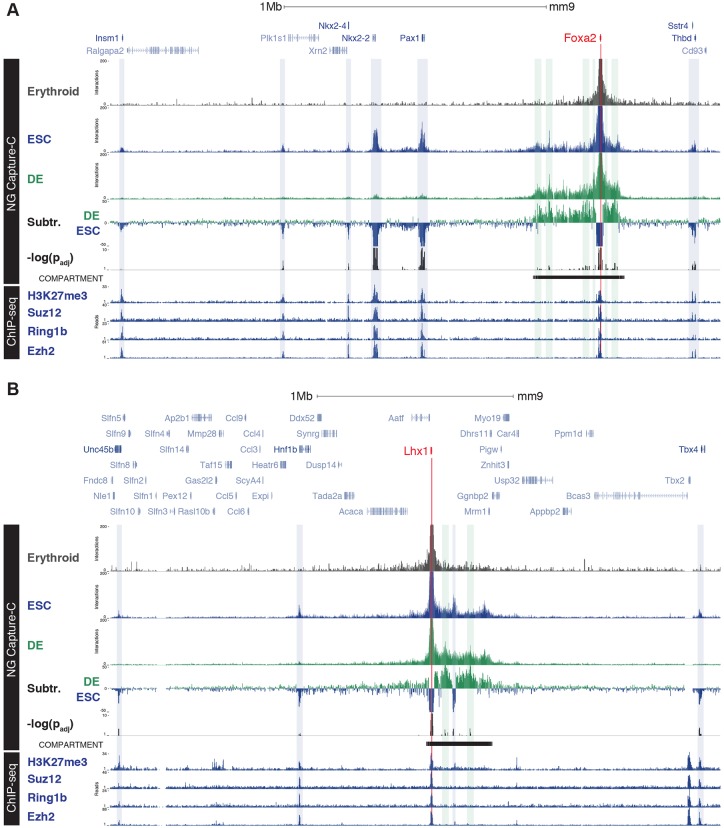
***Foxa2* and *Lhx1* form long-range interactions with polycomb-repressed promoters.** (A,B) NG Capture-C interaction profiles of the *Foxa2* (A) and *Lhx1* (B) promoters from erythrocytes (grey), ESC (blue) and DE (green) with chr2: 146,001,500-148,328,000 (A) and chr11: 82,700,000-85,808,000 (B) shown. Tracks show mean interactions of normalised biological replicates (*n*=3), subtraction of ESCs from DE (Subtr.) and DESeq2 significant differences between DE and ESC [−log(*P*_adj_); *P*≤0.05]. Peaks of the strongest interactions in ESCs (shaded boxes) were manually identified and highlighted. Compartments were determined by boundaries of strong (continuous) promoter interactions. Location of the Polycomb Repressor Complexes components (Ezh2, Suz12, Ring1b) and associated histone modification (H3K27me3) in ESCs are shown (Ku et al., 2008; Mikkelsen et al., 2007).

The forkhead TF Foxa2 and the LIM domain homeobox TF Lhx1 function together with Eomes as master regulators of APS cell fates (Ang and Rossant, 1994; Costello et al., 2015; Perea-Gomez et al., 1999; Shawlot and Behringer, 1995). One possible model is that this pre-configured genomic structure might be a common feature shared by endoderm-specific transcriptional factors (Fig. S7C). As for *Eomes*, Capture-C of the *Foxa2* and *Lhx1* promoters demonstrates localisation within pre-formed compartments (both ∼350 kb) in ESCs, but not in erythrocytes where the genes are inactive (Fig. 6A,B). However, these *Foxa2* and *Lhx1* compartments were found to undergo significant rearrangements during DE differentiation (Fig. 6A,B). Unlike *Eomes*, *Lhx1* and *Foxa2* promoters both make long-range contacts with neighbouring developmental genes lying outside the compartment boundaries in ESCs (Fig. 6A,B). These long-range interactions range from 370 kb to 1.8 Mb in size and are almost entirely specific to gene promoters (Table S3); they are lost as cells acquire a DE fate (Fig. 6A,B).

Both *Foxa2* and *Lhx1* are repressed by polycomb in ESC (Leeb et al., 2010). Examination of published ESC ChIP-seq data-sets for Polycomb components Ezh2, Suz12 (PRC2) and Ring1b (PRC1) (Chen et al., 2008; Ku et al., 2008), as well as the polycomb repressive mark H3K27me3 (Yue et al., 2014), showed they are present at all of the promoters of the adjacent genes with which *Lhx1* and *Foxa2* interact (Fig. 6), suggesting that these genes are present in Polycomb bodies (Pirrotta and Li, 2012). Interestingly, these Polycomb repressive components are also present at the *Eomes* promoter in ESC, but we found no evidence for long-range interactions with gene promoters lying outside the compartment (Fig. S10). Collectively, the results above demonstrate that three essential TFs required for cell fate specification, *Eomes*, *Foxa2* and *Lhx1*, were found to exhibit distinct modes of 3D chromatin organisation during differentiation.

## DISCUSSION

The spatiotemporal expression of key lineage-specifying transcription factors (TF) is tightly controlled during early mouse development to ensure correct cell fate decisions. Interactions of cell type-specific *cis*-acting enhancer elements with gene promoters, within topologically discrete chromatin compartments, directs developmentally regulated patterns of expression (de Laat and Duboule, 2013). Our recent studies demonstrate that the T-box TF Eomes, dynamically expressed in the VE, ExE and PS during gastrulation, acts downstream of the Nodal signalling pathway as an essential master-regulator of the DE and cardiac mesoderm cell lineages. Here, we exploit transgenic reporter assays, targeted deletion and NG Capture-C strategies to investigate the regulatory landscape at the *Eomes* locus.

We demonstrate using gain-of-function experiments that conserved proximal *cis*-regulatory elements, namely the so-called PSE (comprising PSE_a and PSE_b) and the VPE, have the ability to drive reporter activity in the PS, or VE and PS, respectively. The conserved *Eomes* PSE_b region, which represents an archetypal poised developmental enhancer in both human and mouse ESC, was recently shown to be activated upon mesendoderm induction in response to Nodal (Smad2/3, Foxh1) and Wnt (β-cat) signalling pathways (Beyer et al., 2013; Brown et al., 2011; Buecker and Wysocka, 2012; Estarás et al., 2015; Funa et al., 2015; Kartikasari et al., 2013; Kim et al., 2011; Rada-Iglesias et al., 2011). However, surprisingly our targeted deletion experiments demonstrate that this switch enhancer, and the adjacent PSE_a, are dispensable for correct developmentally regulated *Eomes* expression in the early embryo. Moreover, mutant mice that entirely lack this genomic region develop normally and are viable and fertile.

Eomes is required for the maintenance and migration of the AVE (Nowotschin et al., 2013). Additionally, robust expression in the PS is essential for formation of APS progenitors (Arnold et al., 2008a). The present results demonstrate that the VPE activates expression in both the AVE and PS, and makes important functional contributions that govern Eomes activities during gastrulation. We found that removal of this element halves transcriptional output from the locus as assessed *in vitro*. Moreover, *Eomes*^ΔVPE/−^ embryos exhibit pleiotropic tissue defects, due to compromised specification of AVE or APS, that closely resemble those caused by defective Nodal signalling or loss of the Smad2/3/4 co-factor *Foxh1* (Arnold et al., 2008a; Hoodless et al., 2001; Norris et al., 2002; Yamamoto et al., 2001).

Our NG Capture-C experiments revealed that the VPE directly interacts with the *Eomes* promoter in both ESC and DE. Moreover the *Eomes* locus lies within a large pre-formed 3D regulatory chromatin compartment in pluripotent ESCs that is maintained upon differentiation to DE. Thus, activation of the locus occurs in the absence of remodelling long-range interactions. By contrast, previous studies of mouse and human ESC implicate *de novo* enhancer-promoter interactions during DE and mesendoderm differentiation (Estarás et al., 2015; Kartikasari et al., 2013). These inconsistencies probably reflect technical differences because a target-led (one-versus-some) 3C PCR technique was used previously, when compared with the unbiased (one-versus-all) NG Capture-C sequencing approach exploited here.

**Fig. 7.**
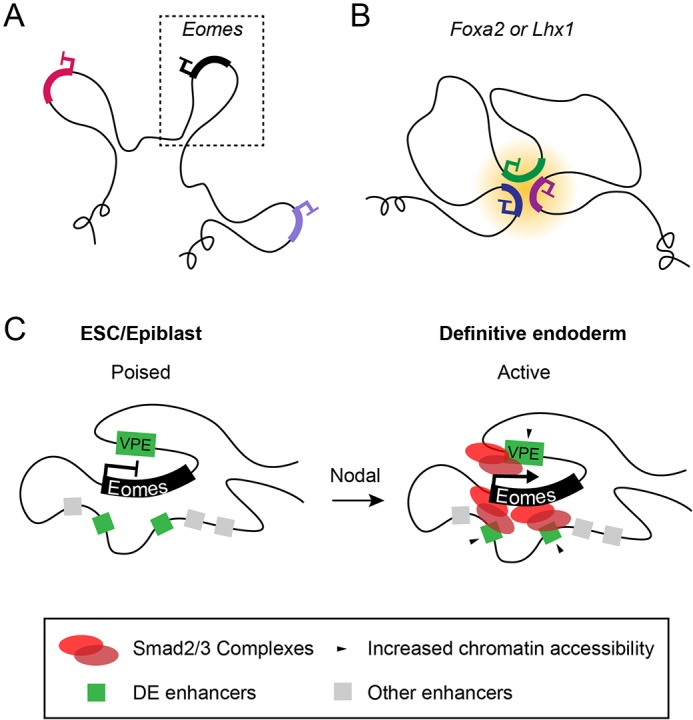
***Eomes*, *Foxa2* and *Lhx1* exhibit distinct modes of 3D chromatin organisation during differentiation.** (A) In ESCs, *Eomes*, *Foxa2* and *Lhx1* are organised into pre-formed chromatin compartments. (B) Unlike *Eomes*, both *Foxa2* and *Lhx1* promoters form extra-compartmental contacts with other polycomb-repressed gene promoters. (C) Model for *Eomes* activation. The poised chromatin architecture at the *Eomes* locus is permissive for rapid transcriptional induction in response to localised Nodal signalling during gastrulation, primarily via enhancer binding of Smad2/3 complexes.

NG Capture-C analysis of the direct Eomes targets *Foxa2* and *Lhx1*, which are known to regulate APS fates, demonstrates they similarly occupy discrete regulatory compartments in transcriptionally silent ESC. However, in contrast to *Eomes*, *Foxa2* and *Lhx1* promoters display contacts with polycomb-associated gene promoters that lie far outside their compartments. These associations are specifically lost during DE differentiation (Fig. 7). Promoter-promoter interactions within ESCs are often occupied by polycomb repressive complexes (PRC) that organise the 3D chromatin structure into polycomb bodies to silence gene expression (Denholtz et al., 2013; Schoenfelder et al., 2015; Sexton et al., 2012; Williamson et al., 2014). These epigenetic barriers are thought to block lineage-specifying gene activation and thus prevent precocious differentiation. We demonstrate here that, in contrast to *Foxa2* and *Lhx1*, the *Eomes* locus exhibits a distinct mode of regulation. Rather, in the absence of polycomb-mediated repressive contacts, the *Eomes* promoter can rapidly respond to dynamic signalling cues during gastrulation (Fig. 7).

Considerable evidence suggests that stable enhancer-promoter interactions within pre-formed chromatin compartments initiate transcription through the release of paused polymerase (de Laat and Duboule, 2013; Ghavi-Helm et al., 2014; Jin et al., 2013; Williamson et al., 2016). We found that promoter-enhancer interactions are relatively stable. However our ATAC-seq experiments reveal significant changes in open chromatin regions during DE differentiation. We identified several candidate enhancers within the *Eomes* compartment that display increased chromatin accessibility and are greatly enriched for Smad2/3 occupancy upon DE differentiation (Yoon et al., 2015). Moreover, we confirm that Smad2/3 is required for *Eomes* activation, as inhibition of receptor-mediated Nodal/Smad2/3 signalling blocks transcription. Smad2/3 associations with the histone demethylase Jmjd3 are known to be required for the activation of Nodal target genes (Dahle et al., 2010; Kartikasari et al., 2013). Jmjd3 activates poised developmental genes by removing promoter-proximal H3K27me3 and releasing paused polymerase (Chen et al., 2012). We propose that the poised chromatin architecture at the *Eomes* locus is permissive for rapid transcriptional induction in response to localised Nodal signalling during gastrulation, primarily via enhancer binding of Smad2/3/Jmjd3 complexes to release promoter-paused polymerase.

The 3C technologies developed over the past two decades have provided important new insights into the regulatory chromatin landscapes that orchestrate tissue-specific transcription. Here, we characterise for the first time *cis*-regulatory elements that activate *Eomes* expression during gastrulation, and describe the higher order chromatin architecture of the locus. We speculate that the pre-formed chromatin compartment and the absence of additional epigenetic safeguards prior to expression facilitates the rapid induction of *Eomes* expression in response to dynamic signalling cues at the onset of gastrulation. However, the stage of embryonic development during which these compartments are established, and later dismantled, remains elusive. Future studies will investigate whether these enhancers and permissive chromatin configuration are tissue invariant and can also control cell type-specific *Eomes* expression governing cell fate decisions at other sites such as the developing cortex, and adult NK and CD8+ T-cell lineages (Arnold et al., 2008b; Gordon et al., 2012; Pearce et al., 2003).

## MATERIALS AND METHODS

### Animals and PCR genotyping

*Eomes*^GFP/+^ (Arnold et al., 2009) and *Foxh1*^+/−^ (Hoodless et al., 2001) strains were genotyped as described. *Eomes*^ΔPSE/+^, *Eomes*^ΔPSE_b/+^, *Eomes*^ΔVPE/+^ and *Eomes*^GFPΔVPE/+^ strains were generated from targeted ESC clones using standard methods (Arnold et al., 2009) (Figs S2-S5, see supplementary Materials and Methods) and maintained on a mixed 129Sv/Ev/C57BL/6 background. To generate PSE.*LacZ* and VPE.*LacZ* transgenic constructs, the 4.6 kb *Hin*cII-*Kpn*I PSE fragment and a 696 bp PCR-amplified VPE sequence (Table S1), were cloned upstream of a hsp68 promoter, *LacZ* cassette and SV40 polyA signal (Sasaki and Hogan, 1996). Zygotes were injected with *Not*I linearised plasmid and transferred into pseudo-pregnant foster females. Embryos were either collected at E6.5-E7.5 or used to establish stable transgenic mouse lines. PCR genotyping primers are listed in Table S1. All animal experiments were performed in accordance with Home Office (UK) regulations and approved by the University of Oxford Local Ethical Committee.

### ESC differentiation

ESC lines were maintained in DMEM (Invitrogen) supplemented with 15% fetal calf serum (Gibco), 1% penicillin/streptomycin (Invitrogen), 0.1 mM 2-mercaptoethanol (Sigma), 1% glutamine (Invitrogen), 1% MEM non-essential amino acids (Gibco), 1 mM sodium pyruvate (Sigma), 1000 U/ml LIF (ESGRO) on gelatin-coated plates.

For analysis of GFP reporter expression, wild-type (CCE), *Eomes*^GFPΔVPE/+^ and *Eomes*^GFP/+^ ESCs were seeded as 10 μl hanging drops (1×10^4^ cells/ml) in the absence of LIF to induce EB formation. After 2 days, EBs were transferred to suspension culture. For SB inhibition experiments, ES cells were seeded in suspension at low density (1×10^4^ cells/ml) in the absence of LIF to form EBs. On day 3, EBs were cultured in the presence or absence of 10 μM SB431542 inhibitor (Tocris). For DE differentiation, ES cells were induced to form EBs in suspension, as described above, but were transferred on day 2 into N2B27 medium (Cellartis) supplemented with 20 ng/ml activin A (R&D systems) and 20 ng/ml EGF (Peprotech) to induce DE differentiation (Morrison et al., 2008). For Capture-C, ChIP-seq and ATAC-seq experiments, EBs were dissociated by incubation with 0.25% trypsin (Gibco) for 3 min at 37°C with constant agitation followed by gentle pipetting to obtain a single cell suspension.

### RNA analysis

RNA was isolated from using Qiashredder homogeniser (Qiagen), RNeasy mini kit (Qiagen) and RNase-Free DNase Set (Qiagen). RNA was reverse transcribed to cDNA using Superscript III First Strand Synthesis System (Life Technologies) and qRT-PCR was carried out in triplicate using SYBR-green kit (Qiagen) on a Rotagene cycler (Qiagen) with primers listed in Table S1. Relative gene expression was normalised to *Gapdh* and calculated as 2^ΔΔCt^.

### *In situ* hybridisation, X-gal staining and immunofluorescence

Whole-mount *in situ* hybridisation was performed according to published protocols (Behringer et al., 2013). *LacZ* activity was visualised using whole-mount X-gal staining as described previously (Behringer et al., 2013). Whole-mount *in situ* hybridisation and X-gal-stained embryos were photographed after clearing in 80% glycerol.

For immunofluorescence, embryos were fixed overnight in 1% PFA. EBs were fixed in 4% PFA for 30 min at room temperature. Samples were washed in 0.1% Triton-X in PBS, permeabilised in 0.5% Triton-X in PBS for 15 min, washed in 0.1% Triton-X in PBS, then blocked in 0.1% Triton-X, 0.2% BSA and 5% donkey serum in PBS for 2 h at room temperature. Samples were incubated with primary antibodies (Table S2) overnight at 4°C, washed, incubated with secondary antibodies or phalloidin AlexaFluor 633 stain (A22284; Invitrogen) in block solution for 2 h at room temperature, counterstained with DAPI and mounted in Vectashield (Vector Laboratories) on chamber slides (LabTek). Images were acquired using an Olympus FV1000 inverted confocal microscope.

### Flow cytometry

Day 4 EBs were incubated in 0.25% trypsin for 5 min at 37°C and dissociated into single cells using a 20-guage needle. FACS analysis was performed using a BD FACSCalibur 4 (BD Biosciences) and data analysed using FlowJo.

### ATAC-seq

Tagmentation and indexing of single cell suspensions of ESC, DE and erythrocytes from phenylhydrazine-treated mice (Davies et al., 2016) was performed as previously described (Buenrostro et al., 2013; Hay et al., 2016). Samples were sequenced using a 75-cycle paired-end kit on the Illumina NextSeq platform.

### ChIP-seq

Single cell suspensions (5×10^6^) were cross-linked in 1% formaldehyde for 15 min at room temperature and processed using standard methods. Briefly, cells were lysed on ice for 20 min (5 mM PIPES, 85 mM KCl and 0.5% Igepal-CA 630), and pelleted nuclei were lysed (50 mM Tris-HCl, 10 mM EDTA and 1% SDS). Sonicated chromatin was incubated overnight with anti-H3K4me3 (2 μl; 07-473; Millipore) and Protein A/G Dynabeads (Invitrogen). Beads were washed with RIPA buffer variants (10 mM Tris-HCl, 1 mM EDTA, 0.5 mM EGTA, 1% Triton X-100, 0.1% SDS and 0.1% sodium deoxycholate) – RIPA, high salt RIPA (500 mM NaCl) and RIPA with 250 mM LiCl – and with TE buffer before RNase A (Roche) and proteinase K (Thermo Fisher) treatment. Phenol-chloroform-extracted DNA was indexed using NebNext Ultra II (New England BioLabs), multiplexed and sequenced using a 75-cycle paired-end kit on the Illumina NextSeq platform.

### ATAC-seq and ChIP-seq analysis

ATAC-seq and ChIP-seq data were analysed as described previously (Hay et al., 2016) using a custom pipeline (http://userweb.molbiol.ox.ac.uk/public/telenius/PipeSite.html). Sequenced reads were aligned using Bowtie to the mm9 build of the mouse genome. Genomic browser tracks were generated from pooled data from multiple replicates and normalised per million mapped reads using a custom Perl script. Peak detection was performed with the MACS2 (Feng et al., 2012). For differential analysis, a union set of peaks for each cell type generated from at least two peak calls per site. Peaks were filtered for high ploidy regions using MIG Viewer (McGowan et al., 2013). CTCF-motifs were identified using the FIMO function of MEME Suite (Bailey et al., 2009; Grant et al., 2011).

### NG Capture-C and analysis

NG Capture-C was performed as described previously (Davies et al., 2016) on single cell suspensions of ESC, DE or erythrocytes. Samples were indexed for multiplexing and co-capture of enhancers or promoters using biotinylated 120-mers (Sigma, IDT) designed with the CapSequm webtool (http://apps.molbiol.ox.ac.uk/CaptureC/cgi-bin/CapSequm.cgi) (Hughes et al., 2014) and pooled to a final concentration of 2.9 nM (Table S4). Captured material was pooled and sequenced using the Illumina NextSeq platform with 150 bp paired-end reads (300 cycle kit, Illumina). Reads were mapped using Capture-C scripts (https://github.com/telenius/captureC/releases), analysed as previously described (Hay et al., 2016), and additionally with FourCSeq (Klein et al., 2015) and DESeq2 (Love et al., 2014).
